# Successful prospective quality improvement programme for the identification and management of patients at risk of sepsis in hospital

**DOI:** 10.1136/bmjoq-2018-000369

**Published:** 2019-06-12

**Authors:** Kevin Gallagher, Nicky Blackwell, Ben Thomas, Matthew Trail, Lorraine Stewart, Ross Paterson

**Affiliations:** 1 Department of Clinical and Surgical Sciences, University of Edinburgh, Edinburgh, UK; 2 Department of Urology, Western General Hospital, Edinburgh, UK; 3 Department of Critical Care, Western General Hospital, Edinburgh, UK

**Keywords:** patient safety, healthcare quality improvement, hospital medicine, quality improvement, sepsis

## Abstract

**Objective:**

This audit aimed to improve the speed and completeness of delivery of treatment to urology patients at risk of sepsis in the hospital.

**Patients and methods:**

Patients were prospectively included if they developed a new-onset systemic inflammatory response syndrome, were reviewed by a doctor who thought this was due to infection and prescribed antibiotics. We measured median time to antibiotic administration (TTABx) as the primary outcome. Factors associated with delays in management were identified, targeted quality improvement interventions implemented and then reaudited.

**Results:**

There were 74 patients in the baseline cohort and 69 following interventions. Median TTABx fell from 3.6 (1.9–6.9) hours to 1.7 (1.0–3.8) p<0.001 hours after interventions. In the baseline cohort, factors significantly associated with a delay in TTABx were: an Early Warning Score less than the medical review trigger level; a temperature less than 38°C; having had surgery versus not. Interventions included: reduced medical review trigger thresholds, education sessions, communication aids, a department-specific sepsis protocol. There were significant improvements in the speed and completeness of sepsis management. Improvements were most marked in postoperative patients. Improvement longevity was achieved through continued work by permanent ward nurse practitioners.

**Conclusion:**

A period of baseline prospective study, followed by tailored quality improvement initiatives, can significantly improve the speed and quality of sepsis management for inpatients on an acute hospital ward.

## Introduction

Sepsis is common in acute inpatient hospital wards. Sepsis has a high-mortality rate.^1^ Research has shown that prompt management of severe sepsis or septic shock reduces mortality.^2–4^

Historically, sepsis was defined as ‘the presence of a systemic inflammatory response syndrome (SIRS) caused by infection’. This definition is now outdated.^5^ However, we know that patients with an SIRS caused by infection are at risk of severe sepsis (18%), septic shock (6%) and death (16%).^6^ Therefore, SIRS is widely used as part of screening tools for patients at risk of sepsis. SIRS is also often used for case identification in sepsis quality improvement work.^7 8^

**Figure 1:**
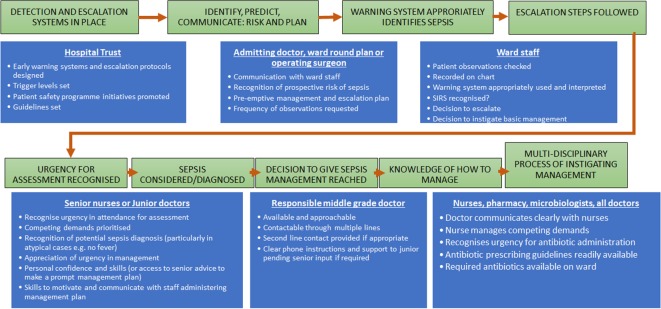
Processes and people involved with the identification and management of patients at risk of sepsis in hospital inpatients. SIRS, systemic inflammatory response syndrome.

When a patient develops sepsis as a hospital inpatient, there are inter-related processes that determine the promptness and completeness of management (figure 1). The Scottish Patient Safety Programme (SPSP) has adopted the ‘sepsis six’ initiative as a means of delivering prompt treatment to patients at risk of sepsis.^9 10^ This initiative recommends that patients with a new-onset SIRS thought to be caused by infection receive the following within 1 hour: (1) blood cultures; (2) lactate measurement; (3) antibiotics; (4) intravenous fluids; (5) oxygen and (6) urine output measurement.

The evidence reporting mortality outcomes in sepsis management, relates to patients with severe sepsis or septic shock.^4 9 10^ In patients with severe sepsis or septic shock, there is evidence that prompt administration of a sepsis management ‘bundle’ reduces mortality. Time to antibiotic administration (TTABx) is individually associated with mortality.^4^ Based on this, we believe that prompt sepsis bundle administration to inpatients at risk of sepsis will improve outcomes.

There has been much attention in the media regarding the under recognition of sepsis and underappreciation of the need for urgency in sepsis management.^11^ The patient safety organisation in Scotland has goals for the management of patients at risk of sepsis. Therefore, we wanted to audit our baseline practice against these guidelines. We intended to use the ‘sepsis six’ bundle as one aspect of this. There are published audit reports about implementing the sepsis six bundle. These are mostly in emergency departments, are often retrospective and may only include qualitative methods to identify reasons for deficiencies.^2 7–9 12^ In contrast, we wanted to undertake a systematic, prospective study of the factors that influence the management of patients at risk of sepsis on a single inpatient ward. We then used combined quantitative and qualitative methods to design a tailored quality improvement programme.

### Problem and setting

At the time of this study, there was no regular audit process for the management of patients at risk of sepsis on our inpatient ward. Anecdotally, there was a problem with delayed recognition and management of patients at risk of sepsis. The ward is a 48-bed acute urology inpatient unit. It has a mix of non-operative, preoperative and postoperative patients. The ward is covered by foundation year 1 (FY1) junior doctors from 8:00 to 22:00 hours. There are once daily ward rounds and contact with urology specialist trainees (ST) via phone. From 22:00 to 8:00 hours, the ward is covered by the general surgical FY1 and core trainee with off-site urology ST on call. Vital signs are measured 4-hourly by ward nurses and were recorded on a local ‘Scottish Early Warning Score’ (SEWS) chart. An SEWS >4 or nursing concern was reason to request a medical review at the time of the baseline study.

## Aims

We aimed to measure baseline performance in the management of patients at risk of sepsis in the urology department, and identify factors associated with management delay or incompleteness. We then aimed to implement targeted quality improvement initiatives. These would be informed by factors identified in the baseline study. The primary objective was to increase the speed of antibiotic delivery to patients at risk of sepsis. We also aimed to increase rate of delivery of the other sepsis 6 steps.

## Methods

Anonymous patient data were recorded prospectively, by twice weekly chart review of all current inpatients, on a 48-bed tertiary urology unit. The baseline study was from November 2012 to June 2013. The postintervention study was from July 2013 to March 2014. Patients were included if they developed a new-onset SIRS, thought to be caused by infection, and were prescribed antibiotics for treatment of this episode. This inclusion criterion was chosen since these patients are at risk of sepsis and would meet our hospital and patient safety agency criteria for urgent administration of the ‘sepsis 6’ within 1 hour. Over the course of the second cycle, improvement initiatives were introduced sequentially over time, as we undertook continuous audit and identified other barriers to improvement that may need addressed.

Patients were excluded if they developed a new-onset SIRS while already receiving antibiotics. The SIRS criteria used were the presence of 2 or more of: heart rate >90 beats per minute, white cell count >12 or less than 4×10^9^/L, temperature>38.0°C, respiratory rate (RR) >20 breaths per minute. The time at which the patient developed a new-onset SIRS as recorded on the observation chart was designated time 0.

The primary outcome measure of this audit was median time to delivery of antibiotics from the time the patient developed a new-onset SIRS. This was chosen because it reflects many processes that we hoped to improve (figure 1). We also examined the rate of delivery of the sepsis six steps.

Patients from the baseline study were then split into two groups: those that received antibiotics faster than the median time (prompt), and those that received antibiotics slower than the median time (delayed). The groups were examined for factors associated with delay to administration of antibiotics including: time of day (8:00 to 18:00 hours vs out of hours); grade of reviewing doctor (FY1 doctor vs more senior); vital signs (heart rate, blood pressure, temperature, RR); white cell count; SEWS; postoperative or non-operative patientand age. Other factors potentially associated with delay to administration of antibiotics were gathered through one to one and group discussions and multidisciplinary training sessions with junior doctors and nurses and fed into improvement initiatives.

Vital signs and Early Warning Scores are not good indicators of the severity of sepsis. However, we hypothesised that these factors would be associated with faster administration of antibiotics since they are used to triage urgency of medical assessment. Therefore, we compared these factors between preintervention and postintervention groups. Factors found to be associated with faster administration of antibiotics on univariable analysis in the baseline study, were then considered potential confounders when determining if interventions were associated with improvements. Therefore, we also undertook regression to adjust for these factors.

### Statistical analysis

Categorical variables were assessed using X^2^ test. Normally distributed continuous variables were analysed using two-tailed independent samples Student’s t-tests. Non-normally distributed continuous variables were expressed as median (25th, 75th) and compared using Mann-Whitney U test. To determine if improvements were due to interventions independent of potential confounding factors, multivariable analysis was performed via linear regression using IBM SPSS statistics V.21.

## Results

In total 143 patients were included, 74 in the first cycle (preintervention) and 69 in the second cycle (postintervention). Patient vital signs and the proportion of patients with an SEWS >4 were not significantly different between groups (table 1).

**Table 1:** Patient characteristics and outcomes preintervention and postintervention

	Preintervention (first cycle)	Postintervention (second cycle)	OR	P value
**Patient characteristics and vital signs**				
N	74	69		
Age	72.0 (56.0–77.0)	73.5 (61.8–80.0)	–	0.16
HR	99.5 (90.8–110.3)	98.0 (87.5–107.0)	–	0.31
HR >90	57 (77.0)	49 (71.0)	–	0.41
Temperature	38.3 (38.0–38.6)	38.3 (38.1–38.7)	–	0.21
Temperature>38°C	56 (75.7)	58 (84.1)	–	0.21
WCC	13.95 (10.9–18.2)	12.7 (10.0–15.7)	–	0.06
WCC >12	52 (70.3)	36 (52.2)	–	0.03
RR	18.0 (16.0–20.0)	18.0 (16.0–21.0)	–	0.92
RR >20	15 (20.3)	19 (27.5)	–	0.31
SBP <100	7	6	–	0.87
SEWS (25th, 75th)	2.0 (1.0–3.0)	2.0 (1.0–3.0)	–	0.92
SEWS >4	14 (18.9)	14 (20.3)	–	0.84
FY1 first reviewer	40 (54.1)	45 (65.2)	–	0.17
Postoperative sepsis	38 (51.4)	44 (63.8)	–	0.13
Non-elective surgery	5 (11.6)	6 (13.0)	–	0.92
Out of hours	40 (54.1)	36 (52.2)	–	0.75
**Outcome measures**				
Median TTAbx	3.6 (1.9–6.9)	1.7 (1.0–3.8)	–	<0.001
Abx <1 hour	2 (2.7)	19 (27.5)	13.68 (3.05–61.37)	<0.001
Abx >3 hours	45 (57.0)	23 (33.3)	0.322 (0.16–0.64)	0.001
Had lactate	12 (16.2)	28 (40.6)	3.53 (1.61–7.72)	0.001
Had blood cultures	43 (58.1)	53 (76.8)	2.39 (1.16–4.93)	0.02
Urine output measured	56 (75.7)	52 (75.4)	0.98	0.97
IVF given	41 (55.4)	43 (62.3)	1.33 (0.68–2.60)	0.4

SEWS, trigger for medical review at time of first cycle=4.

FY1, foundation year 1; HR, heart rate;IVF, intravenous fluid; RR, respiratory rate;SBP, systolic blood pressure;SEWS, Scottish Early Warning Score;TTABx, time to antibiotics from first SIRS;WCC, white cell count.

### Baseline measurement (preintervention, first cycle)

At baseline, median time to antibiotics was 3.6 (1.9–7.2) hours. Factors significantly associated with delay to administration of antibiotics at baseline included an SEWS less than the medical review trigger of 4 (OR 8.4 (95%CI 1.7 to 40.9) p=0.001), temperature less than 38°C (OR 5.02 (95%CI 1.5 to 17.2) p=0.01) and having had surgery versus not (OR 6.4 (95%CI 2.3 to 17.6) p=0.0003) (table 2). Patients who became septic out of hours (18:00 to 8:00) tended to have delayed antibiotic administration compared with in-hours (table 2).

**Table 2:** Factors associated with receiving antibiotics slower than the median time within patients in the first audit cycle

Factors associated with delayed antibiotics in the first cycle			
First cycle	Slower than median	Faster than median	P value
Total	37	37	
Age	73.0 (62.5–76.5)	70.0 (51.0–79.0)	0.86
SEWS score	2.0 (1.0–3.0)	3.0 (1.0–4.0)	**0.01**
SEWS <4	35 (94.6)	25 (67.6)	**0.003**
WCC	12.6 (8.8–15.7)	14.9 (12.4–20.8)	**0.02**
WCC >12	24 (64.9)	28 (75.7)	0.31
HR	100.0 (93.5–110.5)	98.0 (85.0–109.0)	0.73
HR >90	32 (86.5)	25 (67.6)	0.05
SBP	130.0 (113.5–153.0)	125.0 (108.5–146.5)	0.33
SBP <100	2 (5.4)	6 (16.0)	0.13
Temperature	38.1 (37.6–38.5)	38.5 (38.1–38.9)	0.06
Temperature>38°C	23 (62.2)	33 (89.2)	**0.007**
RR	16 (16–20)	18 (16.5–21.5)	**0.01**
RR >20	5 (13.5)	10 (27.0)	0.15
Out of hours	22 (59.5)	18 (48.6)	0.35
Postoperative	27 (73.0)	11 (29.7)	**<0.001**
FY1	19 (51.4)	21 (56.8)	0.64

SEWS, (trigger for medical review at time of first cycle=4).

Bold values indicate statistical significance.

FY1, foundation year 1; HR, heart rate;RR, respiratory rate;SBP, systolic blood pressure;SEWS, Scottish Early Warning Score;WCC, white cell count.

In the baseline study, postoperative patients waited significantly longer to receive antibiotics than non-operative patients (5.3 (2.98, 11.5) hours vs 2.7 (1.5, 4.19) hours p<0.001) (online supplementary table S1). We also found that 60/74 (81%) of patients with a new-onset SIRS caused by infection, had an SEWS less than the medical review trigger of 4. Overall postoperative patients tended to be less likely to have an SEWS >4, were significantly less likely to have an RR >20 and tended to become septic out of hours more often than non-operative patients (online supplementary table S1).

10.1136/bmjoq-2018-000369.supp1 Supplementary data

### Quality improvement work and strategy

A series of interventions, multidisciplinary education sessions, communication tools and new protocols were introduced (online supplementary figure S1). Our findings were presented to the board-level hospital quality improvement team and at a national quality improvement meeting. These presentations contributed to changes in the hospital trust’s observation chart and early warning trigger level (online supplementary figure S1).

10.1136/bmjoq-2018-000369.supp2 Supplementary data

#### Patient safety programme provided materials

The SPSP champion at our hospital (RP, coauthor, consultant anaesthetist) provided us with the sepsis wallet cards as aide memoirs and the sepsis six stickers to help documentation (online supplementary figure S1).

#### Educating and supporting FY1 doctors

FY1 doctors working during the baseline study were informally interviewed by the study team following presentation of the baseline study findings to the department. We learnt that FY1s felt ill equipped to make decisions regarding prescribing antibiotics in post-operative urology patients and would often delay. The baseline study data supported this. To combat this, we introduced urology focused sepsis teaching at all FY1 induction. KG (urology core surgical trainee at the time) attended FY1 induction and delivered these informal sessions. At induction, we also provided a urology department-specific written sepsis protocol. This was developed by KG/ BT and reviewed and approved by the department clinical director and patient safety consultant (RP). This included specific advice on different kinds of postoperative urology patients.

We also put posters in the FY1 doctor room with a break-down of the surgical teams, escalation flow chart and mobile telephone numbers for each of the registrars. We developed the postoperative sepsis communication tool (online supplementary figure S1). This gave the junior doctors clear instructions on managing individual postoperative patients. This was drafted and reviewed by KG, the junior doctors and consultant surgeons. The hospital printing department aided printing the final version.

#### Multidisciplinary educational interventions

To improve teamwork and communication between nurses and doctors, we organised joint, scenario-based education sessions. These involved table top ward plan scenarios and the ‘septris’ online educational game.^13 14^ One of our coauthors (RP) had spent time in quality improvement and patient safety training and thus had training in running such sessions. He ran the first session, simultaneously training KG and NB who ran subsequent sessions. We undertook one of these 1-hour sessions during each of the two main junior doctor cohorts working in the department during the postintervention audit. The main barrier to these sessions was getting ward nurses and junior doctors all together during the busy working day. We gained support of the ward charge nurse to help. She gave permission for the nurses, clinical support workers and junior doctors to attend the ward seminar room for 30 min just before patient lunch time (a generally less-busy time on the ward.) The baseline study data demonstrated common clinical scenarios in which patients had delayed antibiotic administration, for example, the patient with sepsis without a fever. Such scenarios were included in the education sessions based on actual past events. Formal feedback was gathered on proformas and was positive. All felt that attending joint nurse/junior doctor sepsis education was highly beneficial and fostered a cross-professional teamwork approach to the management of these patients.

#### Reviewing and measuring impact

Interventions were introduced over time. We published a monthly rolling audit using the monthly median TTABxx as our outcome (online supplementary figure S2). This allowed assessment of the impact of interventions such as the education sessions. Interventions already undertaken were repeated so that there was a re-enforcement of the message and guidelines over time.

10.1136/bmjoq-2018-000369.supp3 Supplementary data

### Postintervention: second cycle

Following interventions, median TTABx fell from 3.6 (1.9–6.9) hours to 1.7 (1.0–3.8) hours (p<0.001) (table 1). Patients in the preintervention and postintervention groups had similar SEWS, vital signs and demographics (table 1). ‘Postintervention’ was associated with faster delivery of antibiotics compared with ‘preintervention’ independent of SEWS, temperature, white cell count and postoperative or non-operative status on linear regression (p<0.01). The percentage of patients with sepsis waiting more than 3 hours for antibiotics fell significantly from 45/74 (57.0%) to 23/69 (33.3%) p=0.001 (table 1). The proportion of patients receiving antibiotics within an hour increased significantly from 2/74 (2.7%) to 19/69 (27.5%) p<0.001 (table 1). The rate of delivery of all aspects of the sepsis six increased and was statistically significant for lactate measurement and blood cultures (table 1). Among patients with higher Early Warning Scores (SEWS >4), the median time to antibiotics fell from 2.25 (1.5–3.27) to 1.67 (0.92–3.22) hours although not statistically significant (p=0.35). Despite similar vital signs in the preintervention and postintervention groups, the difference in time to administration of antibiotics between postoperative and non-operative patients resolved (online supplementary table S1). The median TTABx in postoperative patients fell from 5.3 (3.0–11.3) hours to 1.70 (1.0–5.0) hours (p<0.001). The proportion of patients with an SIRS caused by infection, that triggered a medical review, increased from 14/74 (19%) to 33/69 (49%) when the SEWS threshold was lowered to 3 (OR=3.93 (1.85–8.31) p<0.001).

## Discussion

This study aimed to study sepsis management processes and performance in a large urology unit. We analysed the baseline data to design targeted interventions, and then used these to make improvements in the time taken to administer antibiotics. We demonstrated that department-specific problems can be identified by prospective baseline study.

Although there are other audits of ‘sepsis bundle’ implementation in the literature, these are retrospective,^7 8^ focused on severe sepsis,^9^ or focused on acute receiving or accident and emergency departments.^2 4 12^ We believe that our study is unique. We prospectively and quantitatively studied practice in a single ward setting. We then analysed the baseline data to identify factors significantly associated with delay to antibiotic administration. Quantitative data were combined with qualitative findings to design targeted improvement interventions.

We found that patients with a lower Early Warning Score, a temperature less than 38°C and those that had had an operation were at highest risk of waiting >3 hours for antibiotics. One reason for this was that the hospital ‘Early Warning Score’ at the time of the audit, was designed to trigger a medical review when the score was >4. In fact, 80% of the patients meeting the safety agency criteria for the sepsis 6 had an SEWS<4. For patients with an SEWS less than the hospital trigger at presentation, anecdotally we saw that no action was taken until there was a further deterioration.

A reason for management improvements after the interventions, was that more patients with an SIRS ‘triggered’ for a medical review, since the hospital trigger was lowered to 3 (after sepsis audit work in the Health Board, including this study). Furthermore, ‘SIRS’ became an additional reason to trigger a medical review. The SIRS criteria were defined and highlighted on the SEWS chart. This fed in to our message, re-enforced in our education sessions, that over-reliance on the Early Warning Scores should be avoided.

### Were the improvements due to our interventions

It seems likely that the improvements observed are due to the interventions that we made. We accept that this was not a trial and the two cohorts are sequential involving different staff members across staff changes, and therefore, this cannot be conclusively determined. However, the baseline and postintervention audit cycles covered periods of 8 and 9 months, respectively, covering at least two different junior staff cohorts in each audit. Patients in the two cohorts were similar in baseline vital signs and on multivariate analysis ‘postintervention’ was independently associated with reduced median time to antibiotic delivery.

### Barriers to improvement

Barriers to prompt management of sepsis emerged during one to one and group discussions. For junior doctors these included apprehension, a lack of knowledge regarding starting antibiotics in postoperative patients, and competing time demands. To combat this, we introduced a postoperative sepsis communication tool for the operation note, developed a urology unit sepsis protocol, gave mobile phone numbers for patient-specific senior doctors and gave urology specific sepsis teaching at junior doctor induction.

Nurse’s main concern was difficulty in getting doctors to attend to review the patient, and in receiving appropriate communication after medical review about what needed done and how urgently. To combat this, we arranged multidisciplinary interactive scenario-based education sessions involving nurses and junior doctors. Liaising with the ward charge nurse allowed us to identify suitable times in the working day to deliver these sessions.

Not all our initiatives worked. To motivate rapid management of septic patients we thought some light-hearted competition between teams on the ward might help. We published a leader board of ‘time to antibiotic’ results for different teams on one occasion. After feedback from nursing staff, we immediately rescinded this idea as it was felt to foster an unhelpful atmosphere and remove focus from the goal (improved patient management). This was an important learning experience that led us to focus more on encouraging a sense of shared patient safety goals as the motivation for improvement.

### Costs and strategic trade-offs

Patients with worse vital signs or higher Early Warning Scores tended to be managed more promptly at baseline. This suggests that there was some triaging of urgency occurring at baseline. One might argue that all we have done is improve the speed of delivery of antibiotics to patients with mild infections, where it will not make any difference. In patients with higher Early Warning Scores, there was still a noticeable improvement in time to antibiotics after interventions. Evidence has shown that an SIRS caused by infection carries a significant risk of progression to severe sepsis and/or septic shock if left untreated. Therefore, prompt management of patients at risk of severe sepsis (SIRS caused by infection) is a goal worth pursuing. This is an accepted framework in quality improvement and patient safety work (eg, deep vein thrombosis prophylaxis prescribing,^15^ intensive care department safety checklists^16^ or the surgical safety checklist.^17^ Striving for a gold standard, protocol-driven approach to management of a problem across the board raises standards overall while improving hard outcomes for a subgroup of patients.

We accept that lowering thresholds for triggering Early Warning Scores and encouraging prompt antibiotic administration could result in over treatment and extra work for junior doctors. This is particularly relevant in postoperative patients where SIRS has many non-infective causes. We believe that if there are clear instructions for the junior doctors and easy access to senior advice, decision-making and management implementation becomes faster and thus less time is spent per patient.

To balance sepsis management initiatives, routine audit of ward *Clostridium difficile* and other resistant organism infections should be carefully monitored. The rate of antibiotic prescribing in postoperative patients should be an additional balancing measure. This is because the aim is not to increase the actual rate of antibiotic prescribing, only the speed of delivery of antibiotics to those that need them. This is an important consideration given the need for antibiotic stewardship.

### Limitations

A limitation of the study is that we did not audit the rate of uptake of the individual interventions such as the sepsis wallet card, the patient note sticker or the postoperative communication tool. Anecdotally, we know that these were used intermittently and preferred by some doctors more than others. One validation of the success of the postoperative sepsis instruction note is that sometime after completion of the audit these postoperative notes had not been replenished in theatre and a consultant called the audit team to ask for more. Intermittent uptake of individual initiatives is to be expected in real life clinical scenarios. However, taken together, the overall awareness of sepsis diagnosis and urgency in management was raised. This likely resulted in faster management of patients overall.

### Longevity of improvements

After the primary study team had completed the audit work, we handed the audit over to a new permanent urology ward nurse practitioner and new urology junior doctor. They recruited prospective junior doctors to help with a simplified rolling sepsis management audit that continued the work began by this study. Their continuing audit subsequently won the Dean’s prize for patient safety in our health board in 2016. Their data showed delivery of antibiotics to 90% of patients at risk of sepsis within an hour. This was more than a year after we completed this study. Thus, finding a motivated member of permanent staff to hand over responsibility to, represents a potential strategy for ensuring safety improvement longevity beyond the period of initial study.

## Conclusion

A systematic prospective assessment followed by targeted interventions can result in significant and sustained improvements in the management of sepsis on an inpatient ward. Initiatives are likely to be unit specific and should be based on a period quantitative and qualitative baseline study, but some broad recommendations can be made from this work:

- A goal-based, local guideline results in improvement in early sepsis management.
- Whether a patient ‘triggers’ the early warning system when they become septic and staff training to recognise patients at risk of sepsis independent of Early Warning Scores is a key factor in prompt delivery of management.
- Specialty-specific guidelines, education and induction in sepsis management are important to empower rotating junior doctors in their decision-making in unfamiliar specialities.
- Pre-emptive patient-specific sepsis management plans from seniors and clear lines of communication between juniors and seniors speeds up management decisions for juniors.
- In the face of a constantly rotating medical staff, permanent members of staff such as nurse practitioners can help ensure safety improvement longevity going forward.
