# Supplementary figures and images for: Successful prospective quality improvement programme for the identification and management of patients at risk of sepsis in hospital
Source: BMJ Open Qual. 2019 Jun 12;8(2):e000369. doi: 10.1136/bmjoq-2018-000369 (PMC6567954; doi:10.1136/bmjoq-2018-000369)

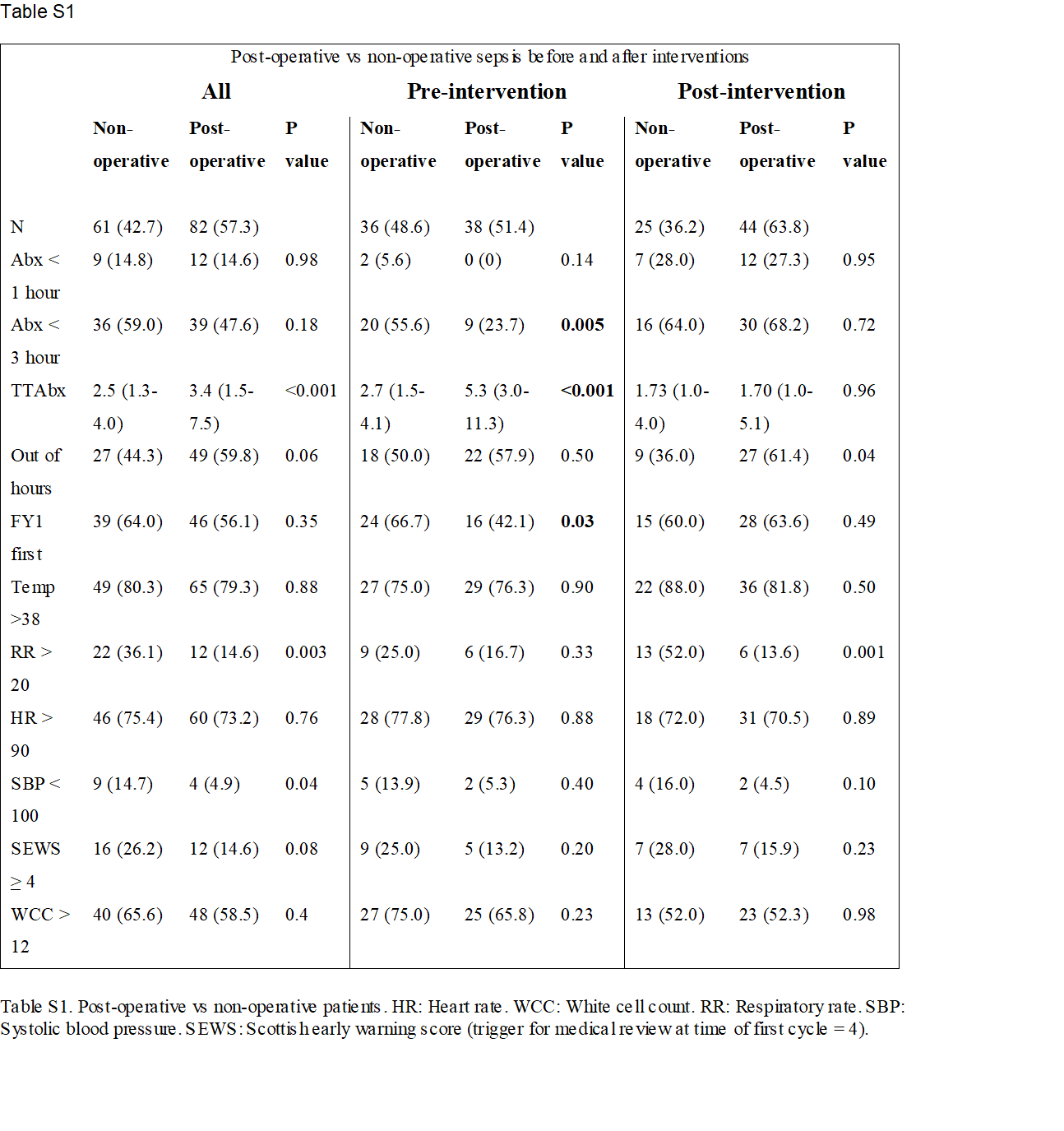

Supplement: Supplementary data [file bmjoq-2018-000369supp001.tif]

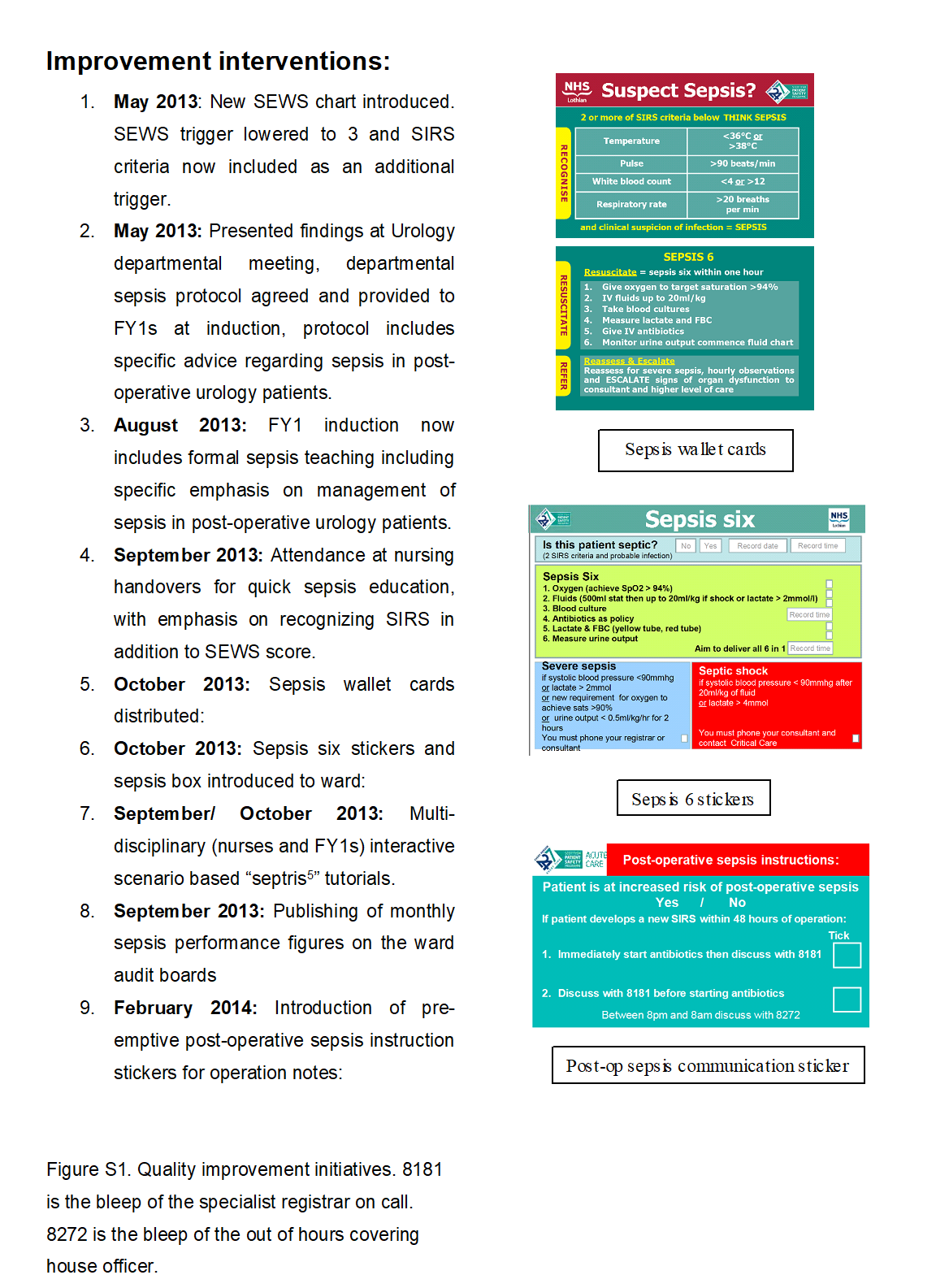

Supplement: Supplementary data [file bmjoq-2018-000369supp002.tif]

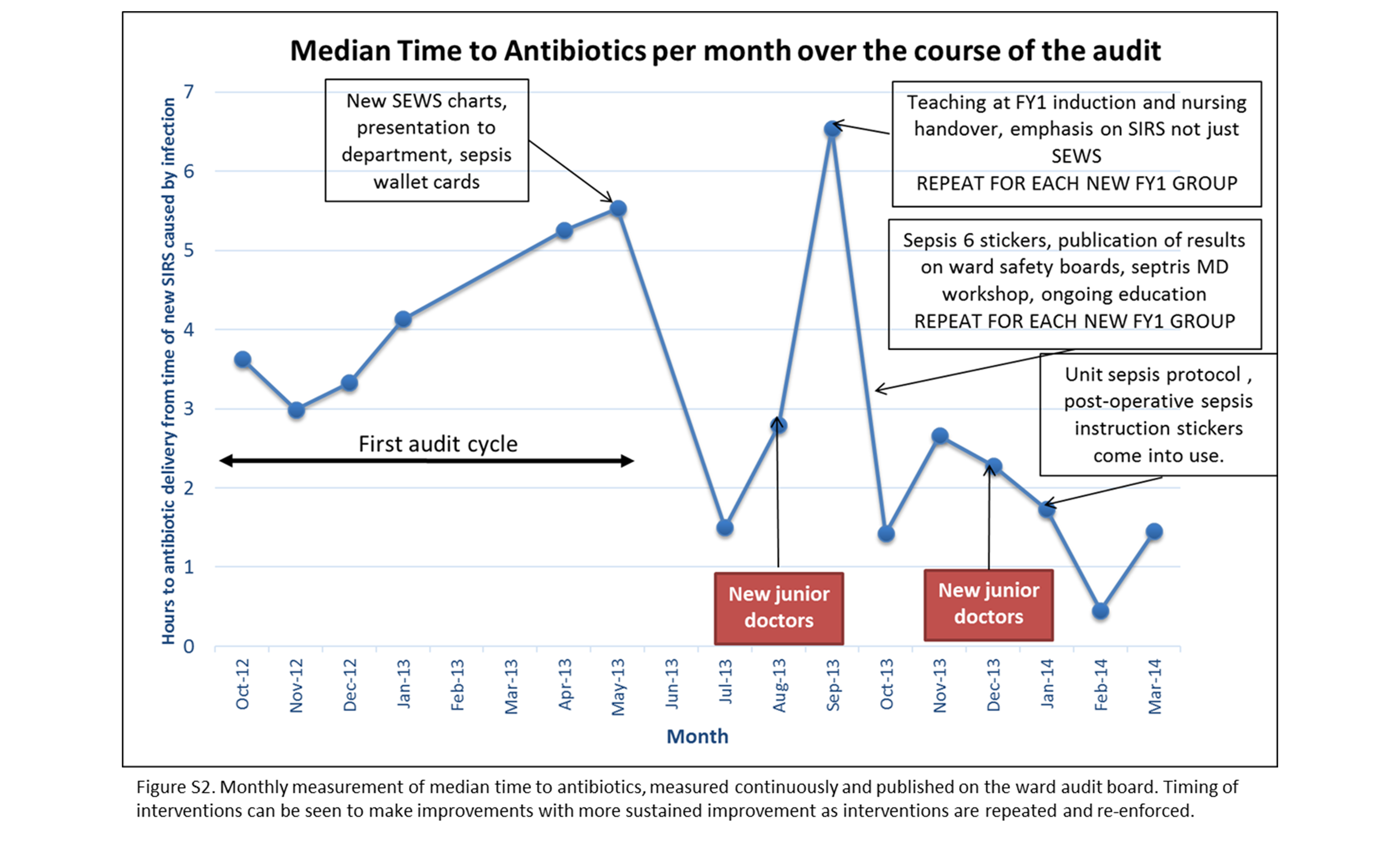

Supplement: Supplementary data [file bmjoq-2018-000369supp003.png]
